# Enhancing the understanding of coinfection outcomes: Impact of natural atypical porcine pestivirus infection on porcine reproductive and respiratory syndrome in pigs

**DOI:** 10.1016/j.virusres.2024.199443

**Published:** 2024-08-01

**Authors:** Holly Hill, David Reddick, Gastón Caspe, Clifford Ramage, David Frew, Mara S. Rocchi, Tanja Opriessnig, Tom N. McNeilly

**Affiliations:** a Moredun Research Institute, Pentlands Science Park, Edinburgh, United Kingdom; b Moredun Scientific, Pentlands Science Park, Edinburgh, United Kingdom; c Estación Experimental Mercedes, Instituto Nacional de Tecnología Agropecuaria (INTA), Mercedes CP 3470, Argentina; d Department of Veterinary Diagnostic and Production Animal Medicine, College of Veterinary Medicine, Iowa State University, Ames, IA, USA

**Keywords:** Atypical porcine pestivirus (APPV), Coinfection, Congenital tremor (CT), Congenital tremor type A-II (CT type A-II), Pigs, Porcine reproductive and respiratory syndrome virus (PRRSV**)**

## Abstract

Atypical porcine pestivirus (APPV) is a novel member of the Pestivirus genus detected in association with congenital tremor (CT) type A-II outbreaks and from apparently healthy pigs, both as singular infection and as part of multi-pathogen infections. 'Classical' pestiviruses are known to cause immunosuppression of their host, which can increase susceptibility to secondary infections, severely impacting health, welfare, and production. To investigate APPV's effect on the host's immune system and characterise disease outcomes, 12 piglets from a natural APPV CT type A-II outbreak were experimentally infected with porcine reproductive and respiratory syndrome virus (PRRSV), a significant porcine pathogen. Rectal temperatures indicating febrile responses, viremia and viral-specific humoral and cellular responses were assessed throughout the study. Pathological assessment of the lungs and APPV-PRRSV co-localisation within the lungs was performed at necropsy. Viral co-localisation and pathological assessment of the lungs (Immunohistochemistry, BaseScope *in situ* hybridisation) were performed post-mortem. APPV status did not impact virological or immunological differences in PRRSV-infected groups. However, significantly higher rectal temperatures were observed in the APPV^+ve^/PRRSV^+ve^ group over four days, indicating APPV increased the febrile response. Significant differences in the lung consolidation of the apical and intermediate lobes were also present, suggesting that APPV co-infection may augment lung pathology.

## Highlights

• A coinfection study was conducted using Atypical porcine pestivirus (APPV).
• APPV in pigs has been characterised recently.
• Rectal temperatures were significantly higher in the coinfected group.
• Coinfected pigs had enhanced lung pathology at post-mortem.
• APPV infection did not significantly impact virological or immunological outcomes.

## 1 Introduction

Multi-pathogen infections are well documented in the swine industry due to intensive husbandry practices that mix young animals during stressful life stages, such as weaning at high stocking densities, with disease outcomes resulting from intricate interactions between coinfecting pathogens and the host's immune system that are sometimes poorly understood (Saade et al., 2020). Atypical porcine pestivirus (APPV), a relatively novel member of the genetically diverse and expanding Pestivirus genus within the *Flaviviridae* family (King et al., 2018; Postel, 2021; Smith, 2017), has been detected concurrently with several significant porcine viral pathogens. APPV is associated with congenital tremors (CT) type A-II in neonatal piglets, often characterised by constant rhythmic tremors. Tremors are non-progressive and vary in severity from localised tremors of the head, flanks or hind limbs to whole body shaking that disrupt the piglet's ability to stand, walk and nurse, leading to starvation (de Groof et al., 2016). The tremors in most cases self-resolve by weaning, however, in severe cases without early-stage farm or veterinary intervention increased mortality rates can occur (de Groof et al., 2016; Pedersen, 2021).

In studies of congenital tremors cases, APPV has been detected as comorbid with both porcine pegivirus (5/67 diagnostic cases) and porcine teschovirus (PTV) (three, three-day-old piglets) (Chen et al., 2019; Possatti et al., 2018). Recently, a study of serum collected from apparently healthy pigs in the US, simultaneously detected APPV in combination with one or more of eight viral pathogens of notable significance for their impact on health and production, including, but not limited to, porcine epidemic diarrhea virus (13/339), swine influenza virus (16/339), porcine circovirus type 2 (23/339), and porcine reproductive and respiratory syndrome virus (PRRSV) (33/339) (Yuan et al., 2021). Co-existence of APPV and several viruses including Getah virus, porcine picobirnavirus, porcine kobuvirus, porcine sapovirus, Po-Circo-like virus, porcine serum-associated circular virus, porcine bocavirus 1, porcine parvovirus 1, porcine parvovirus 5 and porcine circovirus 3 has also been detected through metagenomic analysis in abortion samples (pooled tissue homogenate supernatant from 11 umbilical cord, 1 placenta and 1 aborted piglet) from idiopathic abortion cases in Shandong Provence, China (Sun, 2023).

Pestiviruses are known to have a high economic impact through their ability to interact and suppress the host's immune system, leading to increased susceptibility to secondary infections or overgrowth of commensal and opportunistic pathogens and enhanced the severity and longevity of disease, in both single and dual infections (Baker, 1995; O'Neill et al., 2004; Tarradas et al., 2014); however, this has yet to be fully established for APPV. In this study, we present a coinfection model to assess the effect of a natural APPV infection on a concurrent infection with PRRSV, to investigate the role of APPV as a potential immunosuppressive agent, its role in coinfections and the implications of this for disease outcomes.

Like APPV, PRRSV of the *Arterivirus* genus, family *Arteriviridae*, is a single-stranded positive-sense RNA virus that can be transmitted vertically in utero and horizontally through postnatal infections, affecting young piglets (Done et al., 1996; Feng et al., 2001).

APPV and PRRSV are both globally distributed and detected in both commercial and wild pig populations, with prevalence varying significantly based on pig population, rearing structure, and region (Bálint et al., 2024; Colom-Cadena et al., 2018; de Paz et al., 2015; Postel et al., 2017). A European survey of APPV found genome detection rates ranging from 2.3% in Great Britain to 17.5% in Italy (Postel et al., 2017). Similarly, a 2014 practitioner survey reported PRRSV prevalence in Europe varying from 4% in Russia to 47% in Italy (de Paz et al., 2015).

PRRSV is known to have a significant impact on health, welfare and production, with piglets having increased post-weaning mortality due to clinical signs consistent with respiratory disease, including pyrexia, depression, anorexia, dyspnoea and pneumonia that can increase post-weaning mortality (Haiwick et al., 2018). PRRSV can be found in piglets of a similar age as those with unresolved APPV infections. It is therefore possible that if APPV has similar immunomodulatory effects seen with other pestiviruses, co-infection may influence the immune response to PRRSV, resulting in increased PRRSV-mediated pathology.

To test this hypothesis, 10-week-old piglets naturally infected with APPV were subsequently infected with PRRSV and viral, clinical, immunological, and pathological changes compared to matched control piglets, which were similarly infected with PRRSV but were APPV-negative.

## 2 Materials and methods

### 2.1 Animals

Sixteen mixed-gender large white x Duroc 10-week-old piglets were sourced from a farm with an ongoing congenital tremor outbreak. Male and female piglets were selected from six litters within the same farrowing group, presenting a mixed severity of tremors associated with CT, varying among and within litters. Piglets were assigned either APPV positive with CT clinical signs or APPV negative with no CT clinical signs status based on concurring APPV RT-qPCR results from paired serum and ear notch tissue samples and a clinical evaluation for CT of piglets at 2.5 weeks of age. All piglets were screened for PRRSV by Virotype PRRSV RT-PCR (Indical biosciences) and PRRS X3 Ab ELISA kit (IDEXX). Due to animal welfare concerns regarding the transportation of neonatal piglets with a neurological disorder, the piglets were moved from the farm at nine weeks of age to Moredun Research Institute animal facilities after the resolution of severe tremors, assigned to experimental groups (Table 1) and given a seven-day acclimatisation period before the commencement of the study. The piglets were housed in straw-bedded enclosures with additional toy balls for environmental enrichment. Animals were given adlib access to water troughs and fed pelleted concentrates.

**Table 1:** Summary of study groups based on selected diagnostic criteria at 2.5 weeks of age.

Group	Piglet numbers	APPV clinical signs	APPV nucleic acid		PRRSV nucleic acid	PRRSV antibody	PRRSV infection status
			Serum	Ear notch	Serum	Serum	
Uninfected control	2	No	NVD^1^	NVD^1^	NVD^1^	NAD^2^	No
APPV^-ve^/ PRRSV^+ve^	7	No	NVD^1^	NVD^1^	NVD^1^	NAD^2^	Yes
APPV^+ve^/ PRRSV^+ve^	7	Yes	Positive	Positive	NVD^1^	NAD^2^	Yes

1 NVD (no virus detected),

2 NAD (no antibodies detected).

### 2.2 Viruses

APPV was acquired as a natural infection on the farm, confirmed by APPV RT-qPCR in ear tissue and serum samples and product sequencing (sequence unpublished). A PRRSV species 1 subtype 2 isolate (Genbank accession number: KC714015.1) was propagated using the primary cell line Porcine Alveolar Macrophage (PAM) cells (generated in-house). PAM cells (approx. 1 × 10^5^ cells /mL) were cultured in a 10% Fetal bovine serum (FBS) enriched Roswell Park Memorial Institute (RPMI) at 37°C with 5% CO_2_ for 16 h. For inoculation, media was removed, and the virus was added directly to the flask and incubated for 2 h. Afterwards, fresh media was introduced, and the flask was incubated for 96 h before harvesting. Virus quantification was conducted using median tissue culture infectious dose (TCID_50_) and PRRSV RT-qPCR (Virotype PRRSV 2.0 RT-PCR Kit with in-house plasmid for quantification, Indical Bioscience).

### 2.3 Experimental design

The piglets were assigned into experimental groups based on their clinical and APPV infection status (Table 1). Seven APPV-positive (APPV^+ve^) piglets were assigned to the APPV^+ve^/PRRSV^+ve^ group, and the remaining nine APPV-negative (APPV^-ve^) piglets were assigned to two groups, seven piglets to the APPV^-ve^/ PRRSV^+ve^ and two piglets to the uninfected APPV^-ve^/ PRRSV^-ve^ control group. Intranasal inoculations with 10 mL (5 mL per nostril) of 1.12 × 10^6^ TCID_50_/mL of PRRSV were administered to piglets in the APPV^+ve^/PRRSV^+ve^ and APPV^-ve^/ PRRSV^+ve^ groups on day 0. Rectal temperatures were taken daily from day 1 to 13 of the study, and serum and nasal swab samples were collected on days 0, 1, 3, 5, 7, 10 and 14. At necropsy, the trachea was clamped closed to minimise contamination, and the lungs were removed for visual inspection for consolidation (see Section 2.8) and lavage. The lavage was performed using 50 mL of sterile phosphate-buffered saline (PBS) instilled via the trachea into both lungs. The lungs massaged for 1 min to spread the PBS throughout the lungs before removal of the lavage fluid by inversion over a sterile falcon tube to allow the fluid to drain. Tissue samples were collected from the brain, cervical spinal cord, superficial inguinal lymph node and right cardiac lung lobe for virological evaluation. An additional sample of the right cardiac lung was fixed in 10% neutral buffered formalin (CellPath Ltd.) for pathological examination. The spleen was also collected and washed in Hank's buffered salt solution without calcium or magnesium (HBSS) for splenocyte preparation and lymphocyte re-stimulation assays.

### 2.4 Sample preparation

Serum was derived from whole blood collected in vacutainers containing a silica additive and left to coagulate at 4°C until a visible clot formed. The serum was collected after centrifugation a 2000 x *g* for 10 min at 4°C. Before nucleic acid extraction, the swab samples were processed by vortexing the swab head with 1 mL of sterile PBS. Ear notches were collected using an ear punch tool producing a 1 cm diameter plug which was then shaved and incubated with 0.2 mL of Virotype RLT lysis buffer (QIAGEN) at 65°C for 30 min, then 98°C for an additional 15 min before cooling on ice and centrifuging at 5724 x *g* for 30 s for RT-qPCR.

Tissue samples were homogenised in virus transport medium (APHA Scientific) at a concentration of 0.25 g/mL using the GentleMacs™ dissociator (Miltenyi Biotec), with two cycles of +2000 revolutions per minute (RPM) for four seconds, -2000 RPM for four seconds, +4000 RPM for four seconds then -4000 RPM punctuated by four-second rest intervals. The tissue was centrifuged at 2000 x *g* for 10 min at 4°C, and the supernatant was collected for nucleic acid extraction.

Splenocytes were prepared by homogenising prewashed spleens in stomacher bags before cell straining. Splenocytes were isolated from the spleen homogenate by density gradient centrifugation using Ficoll-Paque® Plus (Sigma Aldrich) and washed twice with PBS. The isolated splenocytes were re-suspended in RPMI Medium with 10% heat-inactivated pestivirus-free FBS, 100 units/mL Penicillin and 100 µg/mL streptomycin at a concentration of 2 × 10^5^ cells/mL for seeding in a lymphocyte re-stimulation assay. Lung samples taken from the right cardiac lobe were fixed at necropsy in 10% neutral buffered formalin for 3 weeks, then dehydrated with alcohol for 24 h and embedded in paraffin. The paraffin-embedded formalin-fixed tissue was cut into 5 µm sections and mounted on slides for further pathological examination.

### 2.5 Virological analysis

Total nucleic acid extraction was performed on all samples except ear tissue using the MagMAX CORE nucleic acid purification kit (Life Technologies) following the manufacturer's instructions in combination with the MagMax express 96 (Thermofisher) using the preset programme (MagMAX_CORE_KF-96_no_heat.bdz).

Nucleic acid extracted from serum swabs and tissues, as well as the lysate from the ear notch tissue preparation, was tested for APPV by RT-qPCR using a previously published primer set (Arruda et al., 2016), including a forward primer (TGCCTGGTATTCGTGGC), a reverse primer (TCATCCCATGTTCCAGAGT) and a modified probe 5′-FAM- CTCCGTCTCCGCGGCTTCTT-BHQ. The assay was performed using the qScript™ XLT One-Step RT-qPCR ToughMix®, Low ROX kit (Quantabio) following the manufacturer's instructions under fast cycling conditions on a QuantStudio5 instrument (Applied Biosystems) with each sample tested in duplicate. Quantification was achieved using a ten-fold serial dilution (3.19 × 10^8^ – 3.19 × 10^1^ copies/ µL) of APPV linearised plasmid designed in-house. Briefly, the APPV PCR product was cloned into pGEM®-T Easy vector (Promega), the DNA plasmid purified using the QIAprep spin miniprep kit (QIAGEN) and linearised by using EcoRI (Promega) restriction enzyme digest. Nucleic acid was also tested by RT-PCR for PRRSV using the Virotype PRRSV RT-PCR kit (Indical Biosciences) following the manufacturer's recommendations. An eight-point, 10-fold serial dilution of linearised PRRSV plasmid was included in each assay for quantification purposes. The PRRSV plasmid was produced using the same methodology as the APPV plasmid.

### 2.6 Serological assays

The humoral response was measured in serum using the PRRS X3 Ab ELISA (IDEXX) to detect anti-PRRSV IgG antibodies. The assay followed the manufacturer's instructions and quantified results as S/P ratios.

### 2.7 Cell-mediated immune assay

PRRSV-specific interferon-γ secreting cells were quantified in splenocytes using Porcine IFN-γ ELISpot^BASIC^ (Mabtech) assays following the manufacturer's instructions. For analysis of the prepared splenocytes, cells were stimulated with concanavalin A (Con A) for 48 h at a final concentration of 5 µg/mL and heat-inactivated PRRSV species 1 subtype virus at 2 5.6 × 10^4^ TCID50/well or media without virus as a media-only control. An AID iSpot ELISpot reader with corresponding AID ELISpot 7.0 Software (AID) was used to inspect and count spot numbers. The results were expressed as the number of spot-forming units (SFU) per 10^6^ cells. Con A and PRRSV-specific responses were reported as the fold-change in SFU/10^6^ cells relative to the media-only control.

### 2.8 Pathology

Each lung lobe was scored for consolidation using a modified scoring system (Jericho and Langford, 1982), which was then converted into a score representing the percentage of lung consolidation relative to the lung lobes’ surface area and volume (Halbur et al., 1995). The left and right apical and cardiac lobes were scored out of 10, the left and right diaphragmatic lobes scored out of 27.5, and the intermediate lobe scored out of 5 with the total lung score out of 100.

Formalin-fixed-paraffin-embedded lung sections were routinely processed with haematoxylin and eosin (H&E, Cellpath Ltd) stain for morphological and interstitial pneumonia evaluation performed using a modified scoring system (Halbur et al., 1995) (Table S1). Immunohistochemistry was performed utilising SDOW- 17, an antibody that detects a nucleocapsid protein of PRRSV (Halbur et al., 1994) (scoring criteria presented in Table S1).

*In situ* hybridisation for APPV was performed on lung sections using BaseScope^TM^ ISH detection reagent kit V2-RED and HybEZ Oven (Biotechne). A viral-specific probe for the detection and quantification of partial NS3 gene was designed in-house using UK APPV strains (ACD Bio, BA-V-APPV-2zz-st and BA-V-APPV-2zz-st1); probes targeting the commonly expressed housekeeping gene peptidyl-prolyl-isomerase-B (*Sus scrofa*-PPIB, 428591) and a bacterial dihydro picolinate reductase (DapB, 310043) were used as the positive and negative control probes respectively. BaseScope *in situ* hybridisation was performed following a modified version of the recommended ACDBio (Biotechne) protocol. Briefly, deparaffinised lung sections were pretreated with hydrogen peroxide for 10 min, washed with distilled water, and submerged in 99°C ±1°C target retrieval buffer for 8 min. In addition to the standard protocol, lung sections were baked for 30 min at 37°C before protease IV treatment. BaseScope V2-RED reagents (AMP 1-8) were applied to the sections following the manufacturer's protocol except for AMP7, which had a prolonged incubation period of 1 h. The slides were counterstained with Gils No 1 haematoxylin (Merck). All slides were digitised using the NanoZoomer ZR and corresponding software NDP.View2 (Hamamatsu) and the slides were used to confirm the presence of APPV (identification of probe signal observed as red dots). To determine quantitative differences between APPV positive and negative groups, five 0.106 mm^2^ areas for each scanned lung section were randomly selected for cell and APPV-specific probe signal counts; these counts were used to determine the number of signals per cell ratio and an overall mean ratio calculated for each lung section.

### 2.9 Statistical analysis

A power calculation was performed to calculate group sizes using previously unpublished PRRSV species 1. Subtype 2 isolate infection data. It was estimated that group sizes of 7 would have been sufficient to detect a 2 log_10_ increase in peak PRRSV viraemia in the APPV-infected piglets with >80% power. The study results were analysed and visualised using GraphPad Prism version 10.1.2. The normality of data was determined via visual inspections of Q-Q plots and a combination of the D'Agostino-Pearson normality test (omnibus K2) (Trujillo-Ortiz and Hernandez-Walls, 2003), Shapiro-Wilk Royston method (Royston, 1995) and Kolmogorov-Smirnov test using Dallal and Wilkinson approximation to Lilliefors’ method (Dallal and Wilkinson, 1986). The normally distributed data was expressed as mean ± standard error of the mean (SEM), and data not normally distributed was expressed as the median ± interquartile range (IRQ). Rectal temperature, viremia, shedding and antibody response data collected as repeated measures throughout the study were analysed by fitting a mixed model, which used a compound symmetry covariance matrix and was fitted using Restricted Maximum Likelihood (REML) with Geisser-Greenhouse correction (Maxwell and Delaney, 2004). Non-parametric Kruskal Wallis tests with post hoc Dunn's test with corrections for multiple comparisons were performed to determine differences in groups for viral loads in tissues, lung pathology including PRRSV-specific Immunohistochemistry (IHC) and PRRSV-specific cellular immune responses (single measures). Normally distributed interstitial pneumonia (IP) scores (single measure) were analysed by one-way ANOVA with post hoc Tukey's HSD testing. P-values < 0.05 were considered statistically significant.

## 3 Results

### 3.1 Observations and exclusions

During the study, although clinical scoring was not implemented, elevated signs of breathing difficulty, lethargy, inappetence, and loss of condition were detected in the APPV^+ve^/PRRSV^+ve^ group at routine health and welfare animal husbandry checks.

Additionally, due to the detection of APPV RNA in tissues at necropsy from two piglets that initially met selection criteria for the APPV^-ve^/PRRSV^+ve^ group, these animals were excluded from the analysis.

### 3.2 Rectal temperature

**Fig. 1:**
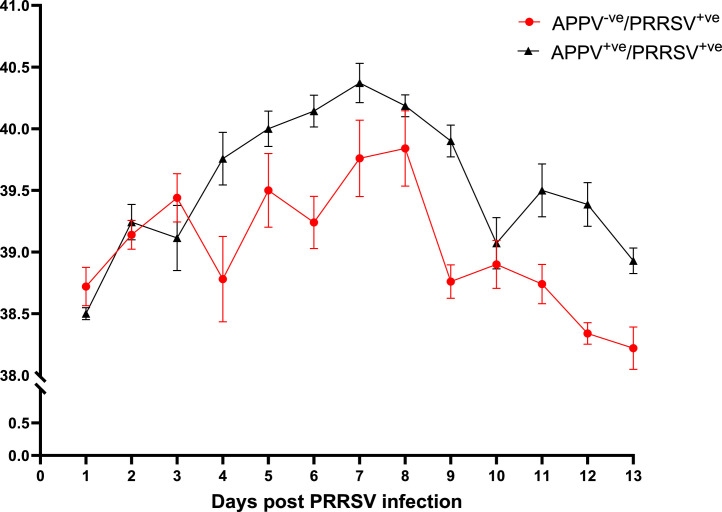
Rectal temperature in APPV-ve/ PRRSV+ve and APPV+ve/ PRRSV+ve pigs following intranasal infection with PRRSV; data represents the mean ± SEM.

The rectal temperature of the APPV^+ve^ /PRRSV^+ve^ group was significantly higher than that of the APPV^-ve^/PRRSV^+ve^ group over the study (P=0.0002). None of the five APPV^-ve^/PRRSV^+ve^ piglets were observed to have persistent fever (≥40°C for ≥3 days) with mean group rectal temperatures of 39.8°C (7-8 DPI) (Fig. 1). In comparison, five of the seven piglets in the APPV^+ve^ /PRRSV^+ve^ group had persistent fever with peak mean temperatures of 40.4°C observed on 7 DPI. The mean rectal temperatures for the APPV^-ve^/PRRSV^+ve^ group were 0.58°C lower than the APPV^-ve^/PRRSV^+ve^ group from 2 DPI -13 DPI.

### 3.3 PRRSV and APPV viral load

#### 3.3.1 Viremia

**Fig. 2:**
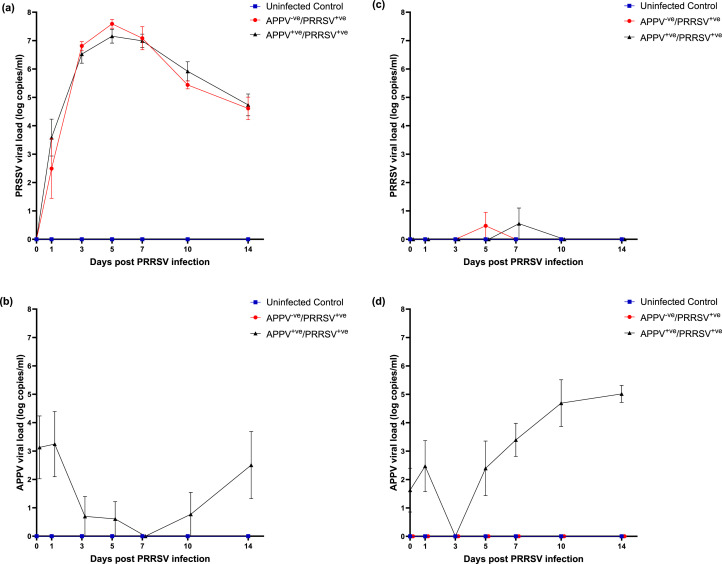
Levels of PRRSV and APPV in serum and nasal swabs. (a) and (c) PRRSV and (b) and (d) APPV viral load in the uninfected control, APPV-ve/ PRRSV+ve and APPV+ve/ PRRSV+ve groups detected in (a) and (b) serum and (c) and (d) nasal swabs. Data represents the mean ± SEM.

There was no significant difference in PRRSV viremia in APPV^-ve^/ PRRSV^+ve^ and APPV^+ve^/ PRRSV^+ve^ piglets. All piglets were PRRSV positive from 1DPI with peak mean viremia of 6.25 × 10^8^ copies/mL and 4.69 × 10^8^ copies/mL detected in the APPV^-ve^ and APPV^+ve^ groups respectively, on day five (Fig. 2a). Inversely, APPV was only detectable in serum from the APPV^+ve^/ PRRSV^+ve^ group from 0 DPI until 3 DPI (1.94—3.12 × 10^5^ copies/mL). The serum viral load decreased from 3DPI to undetectable levels at 7 DPI before increasing to a mean viral load of 3.59 × 10^5^ copies/mL at 14 DPI (Fig. 2b). APPV was not detected in any of the APPV^-ve^/PRRSV^+ve^ piglets, and neither APPV nor PRRSV was detected in the uninfected group during the study.

#### 3.3.2 Nasal shedding

PRRSV was detected in nasal swabs from one APPV^-ve^/PRRSV^+ve^ piglet (6.04 × 10^3^ copies/mL) on 5 DPI and one APPV^+ve^/PRRSV^+ve^ piglet (4.9 × 10^4^ copies/mL) on 7 DPI (Fig. 2c). PRRSV was not detected in nasal swabs from the uninfected group. APPV was consistently detected in nasal swabs from the APPV^+ve^/PRRSV^+ve^ group throughout the study, apart from 3 DPI, where APPV was not detected in any piglet (Fig. 2d). APPV was not detected in nasal swabs from the APPV^-ve^/PRRSV^+ve^ or uninfected control group at any time point.

#### 3.3.3 Viral load in post-mortem tissues and bronchoalveolar lavage

**Fig. 3:**
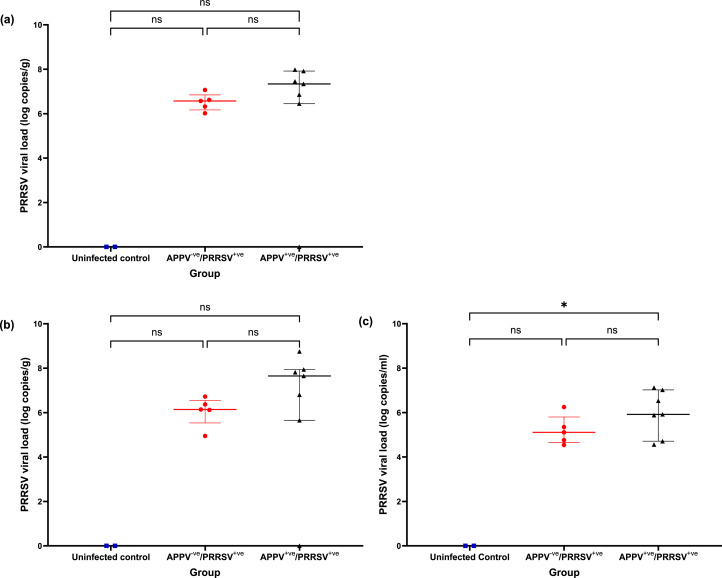
PRRSV viral load in post-mortem tissues and bronchoalveolar lavage. PRRSV viral load in the uninfected control, APPV-ve/ PRRSV+ve and APPV+ve/ PRRSV+ve groups detected in (a) superficial inguinal lymph node, (b) right cardiac lung and (c) bronchoalveolar lavage. The results show individual animals with median, and the interquartile range plotted for each group. Significance (P< 0.05) is indicated on each graph for Dunn's multiple comparisons testing.

PRRSV RNA was detected in all tissues in the APPV^-ve^/ PRRSV^+ve^ and APPV^+ve^/ PRRSV^+ve^ groups at necropsy. The Kruskal analysis determined marginally overall significant differences in PRRSV viral load between the APPV^-ve^ the APPV^+ve^ and uninfected control groups in the superficial inguinal lymph node (H=5.361 P=0.0498), lung (H=5.361, P=0.0498) and bronchoalveolar lavage (H=5.915, P=0.0321). However, the post hoc Dunn's multiple comparisons testing did not identify significant differences in viral loads between the APPV^-ve^/PRRSV^+ve^ and APPV^+ve^/PRRSV^+ve^ groups in the tissues (Z=1.136, P=0.7673) or bronchoalveolar lavage (Z=1.051, P=0.8799) (Fig. 3). The post hoc Dunn's multiple comparisons testing did not find a significant difference between the uninfected controls and either the APPV^-ve/^PRRSV^+ve^ the APPV^+ve/^PRRSV^+ve^ groups in either tissue types (Z=1.377 P=0.5051, and Z=2.267 P=0.0701). In the bronchoalveolar lavage, no significant differences were found between the uninfected controls and the APPV^-ve/^PRRSV^+ve^ group (Z=1.573 P=0.3470). However, there was a significant difference between the uninfected control and APPV^+ve/^PRRSV^+ve^ groups (Z=2.409 P=0.0480). APPV was detected in the superficial inguinal lymph node (7/7), right cardiac lung (7/7), and bronchoalveolar lavage (5/7) from the APPV^+ve^/PRRSV^+ve^ group only, with neither APPV nor PRRSV detected in any of the tissues collected from the uninfected control group.

### 3.4 Immune response to PRRSV

#### 3.4.1 Humoral response to PRRSV

**Fig. 4:**
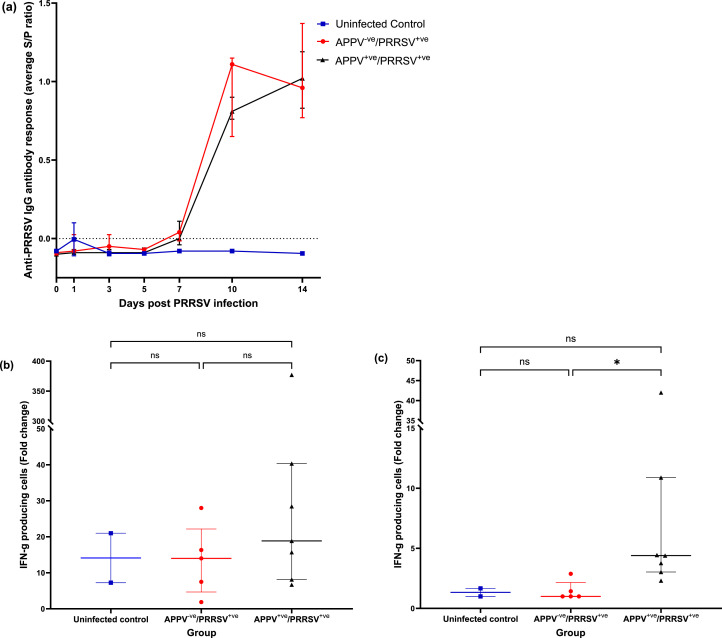
Humoral and cellular response to PRRSV. Immune response to PRRSV infection in the uninfected controls, APPV^-ve^/ PRRSV^+ve^ and APPV^+ve^/ PRRSV^+ve^ groups. (a) Anti-PRRSV IgG antibody levels (mean± SEM). (b) and (c) the number of INF-γ producing cells. The number of INF-γ producing cells determined by ELISpot is calculated as a fold change response to Con A (b) or PRRSV (c), compared to media-only controls. ELISpot data represents individual animals with the median and interquartile range of the group. Significance (P< 0.05) is indicated for Dunn's multiple comparisons testing on each graph.

PRRSV-specific antibody levels were low and generally negative in all piglets in all 3 groups until 10 DPI. On day 10, all piglets in APPV^-ve^/PRRSV^+ve^ and APPV^+ve^/PRRSV^+ve^ groups seroconverted and remained positive until the end of the study at 14 DPI (Fig. 4a). No significant differences in antibody response were observed between the APPV^-ve^/PRRSV^+ve^ and APPV^+ve^/PRRSV^+ve^ groups throughout the study (P> 0.05). The uninfected controls remained seronegative for the duration of the study.

#### 3.4.2 Cellular response to PRRSV

The IFN-γ ELISpot assay was performed on spleen cells following stimulation with either the T cell mitogen ConA or PRRSV antigen to determine both polyclonal and PRRSV-specific T cell responses. No significant difference was found in the number of IFN-γ producing cells between groups after ConA stimulation (Kruskal-Wallis, H=1.494, P=0.5141) (Fig. 4b). However, when stimulated with PRRSV (Fig. 4c), a significant difference in the levels of IFN-γ producing cells was observed between groups (Kruskal-Wallis, H=9.221, P=0.0023). Post hoc Dunn's multiple comparison test indicated the significant difference was between the APPV^-ve^/PRRSV^+ve^ and APPV^+ve^/PRRSV^+ve^ groups (Z=2.789, P=0.0158).

### 3.5 Lung pathology

#### 3.5.1 Gross pathology

Both PRRSV-infected groups presented with visible signs of lung consolidation, although the degree of consolidation was variable across different lobes. Statistical analysis using the Kruskal Wallis test revealed only a significant difference in the weighted lung consolidation scores for both the left and right apical lobes and intermediate lung lobes between the two PPRSV-infected groups (H=6.94, P=0.0262, H=6.894, P=0.0332 and H=6.894, P=0.0332, respectively). The post hoc Dunn's test revealed the consolidation to be significantly higher in the APPV^+ve^/ PRRSV^+ve^ group compared to the APPV^-ve^/ PRRSV^+ve^ group (Z=2.397 P=0.0496) (Table S1). Although no significant difference was indicated in the remaining lobes, a comparative assessment of the consolidation scores demonstrated higher median scores in the APPV^+ve^/ PRRSV^+ve^ group compared to the APPV^-ve^/ PRRSV^+ve^ group across all lung areas, with differences most notable in the Left (APPV^-ve^/ PRRSV^+ve^ 0, APPV^+ve^/ PRRSV^+ve^ 13.8) right (APPV^-ve^/ PRRSV^+ve^ 0, APPV^+ve^/ PRRSV^+ve^ 19.3) and total lung score (APPV^-ve^/ PRRSV^+ve^ 8.3, APPV^+ve^/ PRRSV^+ve^ 44.3). No signs of lung consolidation were visible in any of the lung lobes in the uninfected control group.

#### 3.5.2 Lung histopathology and PRRSV immunohistochemistry

Histopathological evaluation of the lung revealed evidence of interstitial pneumonia (IP) characterised by thickened alveolar septa, an increased presence of pneumocytes type II cells, and inflammatory cells such as macrophages in both PRRSV-infected groups (as indicated by individual Interstitial pneumonia scoring, Table S3). Mild focal peribronchiolar lymphoid hyperplasia was observed in one of the two uninfected control group piglets (IP=1, Table S3), the other showed no signs of interstitial pneumonia. One-way ANOVA found significant differences in the severity of IP scores between groups (F(2,11)=[11.62], P=0.0019) however, Tukey's HSD test found differences between the uninfected control group and APPV^-ve^/PRRSV^+ve^ (P=0.0153, 95% C.I.= [-5.569, -0.6313]) and the uninfected control group and APPV^+ve^/PRRSV^+ve^ (P=0.0014, 95% C.I.=[-6.580, -1.849]), but not between the APPV^-ve^/PRRSV^+ve^ and APPV^+ve^/PRRSV^+ve^ groups (P=0.2338). Additionally, the median IP score of the APPV^+ve^/PRRSV^+ve^ group (5) was higher than that of the APPV^-ve^/PRRSV^+ve^ (4) and uninfected control group (0.5).

**Fig. 5:**
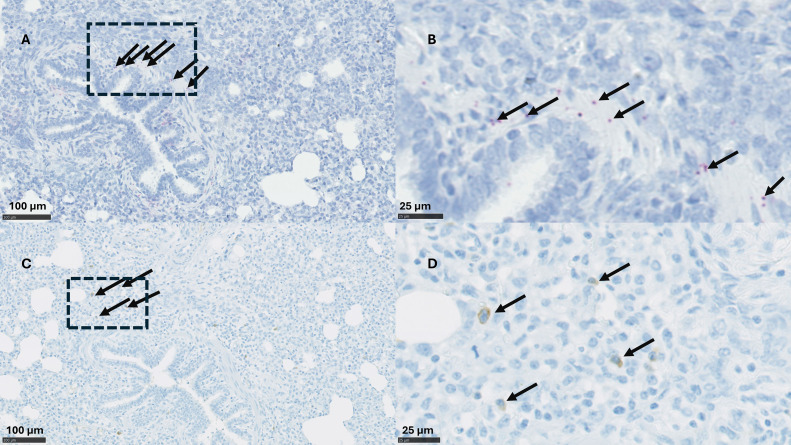
Detection of APPV and PRRSV in sequential right cardiac lung lobe sections from an APPV^+ve^/PRRSV^+ve^ pig. (a-b) APPV detection using BaseScope *in situ* hybridisation indicated by red staining in the smooth muscle of bronchioles; (c-d) PRRSV detection using immunohistochemistry indicated by brown staining in alveolar macrophage-like cells. The arrows indicate positive staining, and the boxes in a and c indicate the area represented in b and d at greater magnification (scale bar: a, c: 100 µm; b, d: 25µm).

Immunohistochemical (IHC) analysis for PRRSV nucleocapsid protein (SDOW- 17) revealed the presence of PRRSV in the lung tissue of both the APPV^-ve^/ PRRSV^+ve^ and APPV^+ve^/ PRRSV^+ve^ groups only. The PRRSV-specific IHC signal was localised in macrophage-like cells located within the alveoli and interstitial space (Fig. 5). Individual IHC scores for each group (Table S3) indicated that animals in the APPV^+ve^/ PRRSV^+ve^ group exhibited a greater number of PRRSV-positive cells (reflected in the higher IHC scores [median=1]) compared to the APPV^-ve^/ PRRSV^+ve^ group (median=0); this increase in IHC scores showed statistical significance in the Kruskal Wallis and post hoc Dunn's tests (H=10.560, P=0.0004 and Z=2.775, P=0.0166 respectively).

#### 3.5.3 APPV detection in the lung using *in situ* hybridisation

APPV-specific staining was successfully detected in the lung tissue of all pigs from the APPV^+ve^/ PRRSV^+ve^ group using BaseScope™ ISH (Fig. 5). The staining pattern exhibited heterogeneity, with the highest proportion of staining observed within the smooth muscle layer of bronchioles and endothelial cells in blood vessels. Kruskal Wallis and post hoc Dunn's tests determined a significantly higher level of APPV staining in the APPV^+ve^/ PRRSV^+ve^ group than the APPV^-ve^/ PRRSV^+ve^, (H=7.844, P0.0066 and Z=2.727, P=0.0192, respectively). Both the APPV^-ve^/ PRRSV^+ve^ and uninfected control groups displayed a minimal level of nonspecific background staining.

## 4 Discussion

Naturally occurring co-infections in pig farms can severely affect pig health and production (Opriessnig et al., 2011; Romeo et al., 2023; Zhao et al., 2021). Pestivirus infections often occur alongside other bacterial and viral pathogens and have been shown to cause immunosuppression in the host species. This immunosuppression is a crucial factor in the development and progression of these infections (Lanyon and Reichel, 2013; Tarradas et al., 2014). While APPV has been identified in coinfections in young pigs (Chen et al., 2019; Possatti et al., 2018), the details of such interactions remain poorly understood, and the immunosuppressive potential of APPV has yet to be fully determined. To the best of the authors' knowledge, this study represents the first attempt to establish an APPV coinfection model specifically designed to investigate the effect of a natural APPV infection on concurrent PRRSV infection and the overall clinical disease outcome for the host.

Our results show a significant and prolonged elevation in rectal temperatures within the APPV^+ve^/PRRSV^+ve^ group compared to the APPV^-ve^/PRRSV^+ve^ group. This finding aligns with a previous study that investigated the co-infection of PRRSV and swIAV, which also reported elevated rectal temperatures (40.0°C-41.4°C) and a longer duration of fever in a higher proportion of the co-infected group (71.5%) compared to the PRRSV-only group (21.5%) (Pomorska-Mól et al., 2020). The prolonged fever in the APPV^+ve^ group indicates that even if APPV has a limited impact on PRRSV replication or the immune response to the virus, it may exacerbate the clinical outcome of PRRSV infection.

Additionally, the elevation in body temperature suggests an inflammatory response triggered by the production of pyrogenic cytokines such as IL-6 in response to viral infection to mitigate the ensuing disease. Increasing body temperature in mammals by 1°C requires a 10-12.5% increase in metabolic rate (Evans et al., 2015), which may contribute to the observed loss of condition in the coinfected group and observed in other studies. Subsequent research should focus on comprehensively understanding the involvement of APPV in the inflammatory response by exploring its impact on cytokine production in relation to weight loss and other clinical outcomes.

Although the main parameter assessed as a metric of clinical outcome was temperature, collateral evidence from routine health and welfare observations performed throughout the study indicated other clinical signs such as breathing difficulty, lethargy, inappetence, and loss of condition, which are commonly observed in PRRSV infection studies (Morgan et al., 2016; Romeo et al., 2023; Stadejek et al., 2017) were also present more noticeably in the APPV^+ve^/PRRSV^+ve^ group.

The pattern of PRRSV viremia in our study was consistent with previous reports (Lunney et al., 2016; Pomorska-Mól et al., 2020; Wesley et al., 2006). Interestingly, the kinetics of APPV viremia showed an inverse relationship with the PRRSV viremia pattern, indicating potential viral interference between APPV and PRRSV observed as a decrease in APPV levels in serum when PRRSV titres increased and then as an increase in APPV titres when PRRSV levels started to decline. The shedding of APPV in nasal swabs also followed a similar pattern as in the sera, further supporting this finding. The detection of PRRSV in nasal swabs was limited in this study. This finding was aligned with other studies that found inconsistent PRRSV detection in nasal swab samples (Charpin et al., 2012; Duan et al., 1997), as factors such as virus species and subtype differences may affect shedding in nasal secretions (Frydas and Nauwynck, 2016; Frydas et al., 2013). Additional sample types and larger sample sizes should be considered in future studies to assess shedding from the respiratory tract. Oral sampling could be advantageous for the dual detection of APPV and PRRSV, as they have successfully detected APPV shedding using this approach (Houston, 2022; Schwarz et al., 2017).

APPV was detected in all pigs in the APPV^+ve^/PRRSV^+ve^ group with comparable viral titres between tissue types, in agreement with previous studies which reported APPV detection by RT-qPCR in all major organs, indicating a systemic viral distribution (de Groof et al., 2016; Muñoz‐González et al., 2017; Postel et al., 2016). PRRSV was detected at similar levels in the bronchoalveolar lavage, lung, and superficial inguinal lymph node of pigs in both the APPV^-ve^/PRRSV^+ve^ and APPV^+ve^/PRRSV^+ve^ groups. This suggests that an active APPV infection may not interfere with the replication of PRRSV.

It should be noted that only tissues from the right cardiac lung lobe were tested for PRRSV viral load, and it may not represent the viral load in the entire lung. However, the detection of high PRRSV titres in the lung and lymphoid tissues is well-documented, and previous studies have demonstrated similar PRRSV viral titres in different lung lobes and bronchoalveolar lavage fluid (Labarque et al., 2000; Morgan et al., 2016; Nazki et al., 2020). The length of the study may have influenced the viral titres detected, and ending the study closer to the viremia peak could have likely resulted in higher viral titres (Labarque et al., 2000; Nazki et al., 2020). Therefore, future investigations should consider different study lengths to determine if differences in viral load between APPV positive and negative PRRSV inoculated groups occur at an earlier point that may have been resolved by the end of the study at day 14.

The humoral immune response to PRRSV was not significantly affected by APPV infection. No notable differences were observed between the APPV^-ve^/PRRSV^+ve^ and APPV^+ve^/PRRSV^+ve^ groups. All pigs infected with PRRSV seroconverted by 10 days post-inoculation and maintained positive antibody levels until the end of the study, which is consistent with previous PRRSV coinfection studies (Duan et al., 1997; Pomorska-Mól et al., 2020), though earlier seroconversion between 8 –9 days post-infection has been reported in PRRSV-only infections (Klinge et al., 2009; Labarque et al., 2000). We cannot exclude that the sampling points chosen for this study for welfare reasons in such young animals may have missed potential seroconversion differences between the groups.

In contrast, the assessment of the cellular immune response to PRRSV by measuring PRRSV-specific IFN-γ responses in spleen cells, a known tissue target for both viruses (Pileri and Mateu, 2016; Postel et al., 2016), found significantly higher IFN-γ responses specific to PRRSV antigen in the APPV^+ve^ group compared to the others. This suggests an enhancement of the T-cell response to PRRSV during co-infection with APPV. This elevated cellular response may have resulted from the high PRRSV viral load identified in the lungs of APPV^+ve^ animals. This could also contribute to the infiltration of inflammatory and immune cells into lung tissue, exacerbating consolidation and pathology in these animals.

The severity of gross pathology observed in the lungs during PRRSV infection can vary depending on various factors such as the strain of PRRSV, breed and age of the animal, and additional environmental stressors (Brockmeier and Lager, 2002; Rossow, 1998; Salguero et al., 2015). Gross pathology examination revealed no discrete lesions except for consolidation in both APPV^-ve^/PRRSV^+ve^ and APPV^+ve^/PRRSV^+ve^ groups. However, significant differences in consolidation scores were observed between APPV^-ve^/PRRSV^+ve^ and APPV^+ve^/PRRSV^+ve^ groups in the apical and intermediate lung lobes. Although overall lung and cardiac or diaphragmatic lobes did not show significant differences, the trend suggested that APPV^+ve^ animals had higher consolidation scores across all lung areas, indicating an impact of APPV on PRRSV lung pathology. This is further supported by the significantly higher IHC scores in the APPV^+ve^ group, which is concomitant with the destruction of alveolar macrophage-like cells that are known to be a cell tropism for PRRSV resulting in targeted pathological changes to the lung (Ruedas-Torres, 2024). Although both PRRSV-infected groups displayed signs of mild to moderate interstitial pneumonia, though not significant, the APPV^+ve^ group displayed a trend towards a higher number of animals with increased IP scores displaying accumulation of intra-alveolar exudate. This, combined with observed increases in the number of type II pneumocytes, may explain the respiratory distress symptoms observed in the more severely affected animals.

Previous studies have demonstrated variations in the distribution of lung pathology following PRRSV infection (Beyer et al., 2000; Chrun et al., 2023; Morgan et al., 2016). Some studies have reported an increase in focal subpleural changes in the diaphragmatic lobes compared to apical, middle, and accessory lobes when pigs were inoculated through the oronasal route (Beyer et al., 2000). Conversely, other studies have found a higher incidence of changes associated with interstitial pneumonia in the apical lung lobes of intranasally inoculated pigs (Morgan et al., 2016). By assessing the distribution of Evan's blue dye as a proxy for viral dispersal within the respiratory tract, Hemmink et al showed significant differences based on the delivery method (Hemmink et al., 2016). Intranasal inoculation primarily targeted the upper respiratory tract and alimentary canal, while aerosol inoculation distributed the dye throughout the upper and lower respiratory tract, encompassing the entire bronchial tree (Hemmink et al., 2016). These findings indicate that intranasal inoculation may not distribute the virus uniformly throughout the lung, leading to a lack of significant pathology in distal lobes. Therefore, future investigations should consider employing a viral inoculation route that ensures equal virus distribution and conducting pathological assessments on all lung areas rather than focusing solely on the cardiac lung lobe, as examined in our study.

Our study also confirmed the presence of APPV in lung tissue, which aligns with previous studies that detected APPV and other pestiviruses in lung tissue using immunohistochemistry and *in situ* hybridisation (Buckley, 2021; Liu et al., 2019; Narita et al., 2000; Nelson et al., 2008), and indicates a specific cell tropism for pestiviruses within the lung. APPV was primarily detected in endothelial cells of blood vessels and bronchiolar smooth muscle cells. Although low levels of APPV were occasionally found in alveoli, they did not co-localise with PRRSV in macrophage-like cells located mainly in areas with interstitial pneumonia. Although the distribution pattern of APPV differed from a previous study, where it was predominantly detected in ciliated bronchioles and weakly in epithelial cells of pulmonary alveoli (Liu et al., 2019). Our finding supports that of another study of a relatively novel Phocoena pestivirus (PhoPeV), which also detected the presence of the pestivirus in bronchiolar smooth muscle cells, alveolar wall, interstitial cells within lung tissue, and smooth muscle cells in arteries, indicating a specific cell tropism (Jo et al., 2019). Detection of APPV in lung tissue using our UK pan APPV strain probes (BA-V-APPV-2zz-st and BA-V-APPV-2zz-st1) further validates the specificity of the BaseScope ISH staining method for virus detection. As APPV and PRRSV have distinct cellular targets, they likely have distinct mechanisms of pathogenicity and immune evasion within the lung tissue. This may explain why differences in PRRSV viral titres of lung tissue are not significantly different between APPV^+ve^ and APPV^-ve^ groups, as they do not directly interact or influence each other's replication within these cells.

## 5 Conclusions

PRRSV and APPV are commonly acquired viruses capable of causing reproductive disease in pigs as single and as a part of complex multiple pathogen infections. PRRSV and APPV can have a significant impact on pig health and welfare, along with substantial economic repercussions for the industry. This study provides valuable insight into the interaction between APPV and PRRSV in a co-infection model. While APPV may not directly enhance or prolong PRRSV infection, it can enhance the clinical disease and lung pathology associated with PRRSV infection. This study's findings highlight the potential immunosuppressive role of APPV and its impact on disease outcomes in coinfections. Additional research using more comprehensive clinical assessments, including behavioural scoring, and measuring changes in weight and respiration rate, is needed to understand the full impact APPV has on the clinical outcome of animals during coinfection. There is also a need to explore the specific interactions and potential synergistic effects between APPV and PRRSV further in the context of co-infection. Understanding the implications of their separate localisation within the lung tissue and their respective cellular targets is crucial for elucidating these viral infections' pathogenesis and clinical impact. Recognising the mechanisms underlying the interaction between APPV and other pathogens is vital for developing effective strategies to control and mitigate the impact of these coinfections in the swine industry. Further investigations are warranted to elucidate the specific immunological mechanisms involved and assess APPV coinfections’ implications with other significant porcine pathogens.

## Ethics statement

All experimental procedures described in this study were approved by the Moredun Research Institute Experiments and Ethics Committee, UK, and were conducted following the legislation of the UK Home Office Project License (reference PFA7E7AD6, Study number E2919, approved 3 June 2019) under the Animals (Scientific Procedures) Act of 1986.

## Funding

This work was supported by Moredun Scientific Ltd. For the purpose of open access, the author has applied a Creative Commons Attribution CC-BY licence to any Author Accepted Manuscript version arising from this submission.

## Author agreement statement

We, the undersigned, declare that this manuscript is original, has not been published before and is not currently being considered for publication elsewhere.

We confirm that the manuscript has been read and approved by all named authors and that no other persons have satisfied the criteria for authorship but are not listed. We further confirm that all of us have approved the order of authors listed in the manuscript.

We understand that the Corresponding Author is the sole contact for the Editorial process. She is responsible for communicating with the other authors about progress, submissions of revisions and final approval of proofs.

## CRediT authorship contribution statement

**Holly Hill:** Writing – original draft, Visualization, Validation, Software, Resources, Methodology, Investigation, Formal analysis, Data curation, Conceptualization. **David Reddick:** Resources, Methodology, Investigation, Conceptualization. **Gastón Caspe:** Writing – review & editing, Visualization, Methodology, Investigation. **Clifford Ramage:** Methodology, Investigation. **David Frew:** Methodology, Investigation. **Mara S. Rocchi:** Writing – review & editing, Supervision, Resources, Methodology, Investigation, Conceptualization. **Tanja Opriessnig:** Writing – review & editing, Supervision, Resources, Project administration, Methodology, Investigation, Data curation, Conceptualization. **Tom N. McNeilly:** Writing – review & editing, Supervision, Resources, Project administration, Methodology, Investigation, Funding acquisition, Conceptualization.

## Declaration of competing interest

The authors declare that they have no known competing financial interests or personal relationships that could have appeared to influence the work reported in this paper.

## Data Availability

Data will be made available on request.

## Appendix Supplementary materials

Image, application 1

## References

[bib62] Arruda B.L., Arruda P.H., Magstadt D.R., Schwartz K.J., Dohlman T., Schleining J.A., Patterson A.R., Visek C.A., Victoria J.G. (2016). Identification of a Divergent Lineage Porcine Pestivirus in Nursing Piglets with Congenital Tremors and Reproduction of Disease following Experimental Inoculation. PloS one.

[bib0001] Baker J.C. (1995). The clinical manifestations of bovine viral diarrhea infection. Vet. Clin. N. Am. Food Anim. Pract..

[bib0002] Bálint Á.C.S., Nemes I., Bijl H., Szabó I. (2024). Investigation of PRRS virus infection in hungarian wild boar populations during its eradication from domestic pig herds. Animals.

[bib0003] Beyer J., Fichtner D., Schirrmeier H., Polster U., Weiland E., Wege H. (2000). Porcine reproductive and respiratory syndrome virus (PRRSV): kinetics of infection in lymphatic organs and lung. J. Vet. Med. B Infect. Dis. Vet. Public Health.

[bib0004] Brockmeier S.L., Lager K.M. (2002). Experimental airborne transmission of porcine reproductive and respiratory syndrome virus and Bordetella bronchiseptica. Vet. Microbiol..

[bib0005] Buckley A.C., Falkenberg S.M., Palmer M.V., Arruda P.H., Magstadt D.R., Schwartz K.J., Gatto I.R., Neill J.D., Arruda B.L. (2021). Distribution and persistence of atypical porcine pestivirus in experimentally inoculated pigs. J. Vet. Diagn. Investig..

[bib0006] Charpin C., Mahé S., Keranflec'h A., Belloc C., Cariolet R., Le Potier M.F., Rose N. (2012). Infectiousness of pigs infected by the Porcine Reproductive and Respiratory Syndrome virus (PRRSV) is time-dependent. Vet. Res..

[bib0007] Chen F., Knutson T.P., Braun E., Jiang Y., Rossow S., Marthaler D.G. (2019). Semi-quantitative duplex RT-PCR reveals the low occurrence of Porcine Pegivirus and Atypical Porcine Pestivirus in diagnostic samples from the United States. Transbound. Emerg. Dis..

[bib0008] Chrun T., Maze E.A., Roper K.J., Vatzia E., Paudyal B., McNee A., Martini V., Manjegowda T., Freimanis G., Silesian A., Polo N., Clark B., Besell E., Booth G., Carr B.V., Edmans M., Nunez A., Koonpaew S., Wanasen N., Graham S.P., Tchilian E. (2023). Simultaneous co-infection with swine influenza A and porcine reproductive and respiratory syndrome viruses potentiates adaptive immune responses. Front. Immunol..

[bib0009] Colom-Cadena A., Ganges L., Muñoz-González S., Castillo-Contreras R., Bohórquez J.A., Rosell R., Segalés J., Marco I., Cabezon O. (2018). Atypical porcine pestivirus in wild boar (Sus scrofa), Spain. Vet. Rec..

[bib0010] Dallal G.E., Wilkinson L. (1986). An analytic approximation to the distribution of Lilliefors's test statistic for normality. Am. Stat..

[bib0011] de Groof A., Deijs M., Guelen L., van Grinsven L., van Os-Galdos L., Vogels W., Derks C., Cruijsen T., Geurts V., Vrijenhoek M., Suijskens J., van Doorn P., van Leengoed L., Schrier C., van der Hoek L. (2016). Atypical porcine pestivirus: a possible cause of congenital tremor type A-II in newborn piglets. Viruses.

[bib0012] de Paz X.V.D., Duran C.O., Angulo J. (2015). Proceedings of the European Symposium of Porcine Health Management.

[bib0013] Done S.H., Paton D.J., White M.E. (1996). Porcine reproductive and respiratory syndrome (PRRS): a review, with emphasis on pathological, virological and diagnostic aspects. Br. Vet. J..

[bib0014] Duan X., Nauwynck H.J., Pensaert M.B. (1997). Virus quantification and identification of cellular targets in the lungs and lymphoid tissues of pigs at different time intervals after inoculation with porcine reproductive and respiratory syndrome virus (PRRSV). Vet. Microbiol..

[bib0015] Evans S.S., Repasky E.A., Fisher D.T. (2015). Fever and the thermal regulation of immunity: the immune system feels the heat. Nat. Rev. Immunol..

[bib0016] Feng W., Laster S.M., Tompkins M., Brown T., Xu J.S., Altier C., Gomez W., Benfield D., McCaw M.B. (2001). In utero infection by porcine reproductive and respiratory syndrome virus is sufficient to increase susceptibility of piglets to challenge by Streptococcus suis type II. J. Virol..

[bib0017] Frydas I.S., Nauwynck H.J. (2016). Replication characteristics of eight virulent and two attenuated genotype 1 and 2 porcine reproductive and respiratory syndrome virus (PRRSV) strains in nasal mucosa explants. Vet. Microbiol..

[bib0018] Frydas I.S., Verbeeck M., Cao J., Nauwynck H.J. (2013). Replication characteristics of porcine reproductive and respiratory syndrome virus (PRRSV) European subtype 1 (Lelystad) and subtype 3 (Lena) strains in nasal mucosa and cells of the monocytic lineage: indications for the use of new receptors of PRRSV (Lena). Vet. Res..

[bib0019] Haiwick G., Hermann J., Roof M., Fergen B., Philips R., Patterson A. (2018). Examination of viraemia and clinical signs after challenge with a heterologous PRRSV strain in PRRS Type 2 MLV vaccinated pigs: a challenge-dose study. PLoS ONE.

[bib0020] Halbur P.G., Andrews J.J., Huffman E.L., Paul P.S., Meng X.J., Niyo Y. (1994). Development of a streptavidin-biotin immunoperoxidase procedure for the detection of porcine reproductive and respiratory syndrome virus antigen in porcine lung. J. Vet. Diagn. Investig..

[bib0021] Halbur P.G., Paul P.S., Frey M.L., Landgraf J., Eernisse K., Meng X.J., Lum M.A., Andrews J.J., Rathje J.A. (1995). Comparison of the pathogenicity of two US porcine reproductive and respiratory syndrome virus isolates with that of the lelystad virus. Vet. Pathol..

[bib0022] Hemmink J.D., Morgan S.B., Aramouni M., Everett H., Salguero F.J., Canini L., Porter E., Chase-Topping M., Beck K., Loughlin R.M., Carr B.V., Brown I.H., Bailey M., Woolhouse M., Brookes S.M., Charleston B., Tchilian E. (2016). Distinct immune responses and virus shedding in pigs following aerosol, intra-nasal and contact infection with pandemic swine influenza A virus, A(H1N1)09. Vet. Res..

[bib0023] Houston G.E., Jones C.K., Woodworth J.C., Palinski R., Paulk C.B., Petznick T., Gebhardt J.T. (2022). Detection and investigation of atypical porcine pestivirus in a swine production system. Front. Vet. Sci..

[bib0024] Jericho K.W., Langford E.V. (1982). Aerosol vaccination of calves with pasteurella haemolytica against experimental respiratory disease. Can. J. Comp. Med..

[bib0025] Jo W.K., van Elk C., van de Bildt M., van Run P., Petry M., Jesse S.T., Jung K., Ludlow M., Kuiken T., Osterhaus A. (2019). An evolutionary divergent pestivirus lacking the N(pro) gene systemically infects a whale species. Emerg. Microbes Infect..

[bib0026] King A.M.Q., Lefkowitz E.J., Mushegian A.R., Adams M.J., Dutilh B.E., Gorbalenya A.E., Harrach B., Harrison R.L., Junglen S., Knowles N.J., Kropinski A.M., Krupovic M., Kuhn J.H., Nibert M.L., Rubino L., Sabanadzovic S., Sanfaçon H., Siddell S.G., Simmonds P., Varsani A., Zerbini F.M., Davison A.J. (2018). Changes to taxonomy and the International Code of virus classification and nomenclature ratified by the international committee on taxonomy of viruses (2018). Arch. Virol..

[bib0027] Klinge K.L., Vaughn E.M., Roof M.B., Bautista E.M., Murtaugh M.P. (2009). Age-dependent resistance to Porcine reproductive and respiratory syndrome virus replication in swine. Virol. J..

[bib0028] Labarque G.G., Nauwynck H.J., Van Reeth K., Pensaert M.B. (2000). Effect of cellular changes and onset of humoral immunity on the replication of porcine reproductive and respiratory syndrome virus in the lungs of pigs. J. Gen. Virol..

[bib0029] Lanyon S.R., Reichel M.P. (2013). Understanding the impact and control of bovine viral diarrhoea in cattle populations. Springer Sci. Rev..

[bib0030] Liu J., Li Z., Ren X., Li H., Lu R., Zhang Y., Ning Z. (2019). Viral load and histological distribution of atypical porcine pestivirus in different tissues of naturally infected piglets. Arch. Virol..

[bib0031] Lunney J.K., Fang Y., Ladinig A., Chen N., Li Y., Rowland B., Renukaradhya G.J. (2016). Porcine reproductive and respiratory syndrome virus (PRRSV): pathogenesis and interaction with the immune system. Annu. Rev. Anim. Biosci..

[bib0032] Maxwell S.E., Delaney H.D. (2004).

[bib0033] Morgan S.B., Frossard J.P., Pallares F.J., Gough J., Stadejek T., Graham S.P., Steinbach F., Drew T.W., Salguero F.J. (2016). Pathology and virus distribution in the lung and lymphoid tissues of pigs experimentally inoculated with three distinct type 1 PRRS virus isolates of varying pathogenicity. Transbound. Emerg. Dis..

[bib0034] Muñoz-González S., Canturri A., Pérez-Simó M., Bohórquez J., Rosell R., Cabezón O., Segalés J., Domingo M., Ganges L. (2017). First report of the novel atypical porcine pestivirus in Spain and a retrospective study. Transbound. Emerg. Dis..

[bib0035] Narita M., Kawashima K., Kimura K., Mikami O., Shibahara T., Yamada S., Sakoda Y. (2000). Comparative immunohistopathology in pigs infected with highly virulent or less virulent strains of hog cholera virus. Vet. Pathol..

[bib0036] Nazki S., Khatun A., Jeong C.G., Mattoo S.u.S., Gu S., Lee S.I., Kim S.C., Park J.H., Yang M.S., Kim B., Park C.K., Lee S.M., Kim W.I. (2020). Evaluation of local and systemic immune responses in pigs experimentally challenged with porcine reproductive and respiratory syndrome virus. Vet. Res..

[bib0037] Nelson D.D., Dark M.J., Bradway D.S., Ridpath J.F., Call N., Haruna J., Rurangirwa F.R., Evermann J.F. (2008). Evidence for persistent Bovine viral diarrhea virus infection in a captive mountain goat (Oreamnos americanus). J. Vet. Diagn. Investig..

[bib0038] O'Neill R.G., O'Connor M., O'Reilly P.J. (2004). A survey of antibodies to pestivirus in sheep in the Republic of Ireland. Ir. Vet. J..

[bib0039] Opriessnig T., Giménez-Lirola L.G., Halbur P.G. (2011). Polymicrobial respiratory disease in pigs. Anim. Health Res. Rev..

[bib0040] Pedersen K., Kristensen C.S., Strandbygaard B., Bøtner A., Rasmussen T.B. (2021). Detection of atypical porcine pestivirus in piglets from Danish sow herds. Viruses.

[bib0041] Pileri E., Mateu E. (2016). Review on the transmission porcine reproductive and respiratory syndrome virus between pigs and farms and impact on vaccination. Vet. Res..

[bib0042] Pomorska-Mól M., Podgórska K., Czyżewska-Dors E., Turlewicz-Podbielska H., Gogulski M., Włodarek J., Łukomska A. (2020). Kinetics of single and dual simultaneous infection of pigs with swine influenza A virus and porcine reproductive and respiratory syndrome virus. J. Vet. Intern. Med..

[bib0043] Possatti F., Headley S.A., Leme R.A., Dall Agnol A.M., Zotti E., de Oliveira T.E.S., Alfieri A.F., Alfieri A.A (2018). Viruses associated with congenital tremor and high lethality in piglets. Transbound. Emerg. Dis..

[bib0044] Postel A., Hansmann F., Baechlein C., Fischer N., Alawi M., Grundhoff A., Derking S., Tenhündfeld J., Pfankuche V.M., Herder V., Baumgärtner W., Wendt M., Becher P. (2016). Presence of atypical porcine pestivirus (APPV) genomes in newborn piglets correlates with congenital tremor. Sci. Rep..

[bib0045] Postel A., Meyer D., Cagatay G.N., Feliziani F., De Mia G.M., Fischer N., Grundhoff A., Milićević V., Deng M.C., Chang C.Y., Qiu H.J., Sun Y., Wendt M., Becher P. (2017). High abundance and genetic variability of atypical porcine pestivirus in pigs from Europe and Asia. Emerg. Infect. Dis. J..

[bib0046] Postel A.S., Smith D.B., Becher P. (2021). Proposed update to the taxonomy of pestiviruses: eight additional species within the genus pestivirus, Family Flaviviridae. Viruses.

[bib0047] Romeo C., Parisio G., Scali F., Tonni M., Santucci G., Maisano A.M., Barbieri I., Boniotti M.B., Stadejek T., Alborali G.L. (2023). Complex interplay between PRRSV-1 genetic diversity, coinfections and antimicrobial use influences performance parameters in post-weaning pigs. Vet. Microbiol..

[bib0048] Rossow K.D. (1998). Porcine reproductive and respiratory syndrome. Vet. Pathol..

[bib0049] Royston P. (1995). Remark AS R94: a remark on algorithm AS 181: the W-test for normality. J. R. Stat. Soc. Ser. C (Appl. Stat.).

[bib0050] Ruedas-Torres I., Sánchez-Carvajal J.M., Salguero F.J., Pallarés F.J., Carrasco L., Mateu E., Gómez-Laguna J., Rodríguez-Gómez I.M. (2024). The scene of lung pathology during PRRSV-1 infection. Front. Vet. Sci..

[bib0051] Saade G., Deblanc C., Bougon J., Marois-Créhan C., Fablet C., Auray G., Belloc C., Leblanc-Maridor M., Gagnon C.A., Zhu J., Gottschalk M., Summerfield A., Simon G., Bertho N., Meurens F. (2020). Coinfections and their molecular consequences in the porcine respiratory tract. Vet. Res..

[bib0052] Salguero F.J., Frossard J.P., Rebel J.M., Stadejek T., Morgan S.B., Graham S.P., Steinbach F. (2015). Host-pathogen interactions during porcine reproductive and respiratory syndrome virus 1 infection of piglets. Virus Res..

[bib0053] Schwarz L., Riedel C., Högler S., Sinn L.J., Voglmayr T., Wöchtl B., Dinhopl N., Rebel-Bauder B., Weissenböck H., Ladinig A. (2017). Congenital infection with atypical porcine pestivirus (APPV) is associated with disease and viral persistence. Vet. Res..

[bib0054] Smith D.B., Meyers G., Bukh J., Gould E.A., Monath T., Scott Muerhoff A., Pletnev A., Rico-Hesse R., Stapleton J.T., Simmonds P., Becher P. (2017). Proposed revision to the taxonomy of the genus Pestivirus, family Flaviviridae. J. Gen. Virol..

[bib0055] Stadejek T., Larsen L.E., Podgórska K., Bøtner A., Botti S., Dolka I., Fabisiak M., Heegaard P.M.H., Hjulsager C.K., Huć T., Kvisgaard L.K., Sapierzyński R., Nielsen J. (2017). Pathogenicity of three genetically diverse strains of PRRSV Type 1 in specific pathogen free pigs. Vet. Microbiol..

[bib0056] Sun X., Zhang Q., Shan H., Cao Z., Huang J. (2023). Genome characteristics of atypical porcine pestivirus from abortion cases in Shandong Province, China. Virol. J..

[bib0057] Tarradas J., de la Torre M.E., Rosell R., Perez L.J., Pujols J., Muñoz M., Muñoz I., Muñoz S., Abad X., Domingo M., Fraile L., Ganges L. (2014). The impact of CSFV on the immune response to control infection. Virus Res..

[bib0058] Trujillo-Ortiz, A., Hernandez-Walls, R. (2003). DagosPtest: D'Agostino-Pearson's K2 test for assessing normality of data using skewness and kurtosis*.* [Matlab file].

[bib0059] Wesley R.D., Lager K.M., Kehrli M.E. (2006). Infection with Porcine reproductive and respiratory syndrome virus stimulates an early gamma interferon response in the serum of pigs. Can. J. Vet. Res..

[bib0060] Yuan F., Feng Y., Bai J., Liu X., Arruda B., Anbalagan S., Peddireddi L. (2021). Genetic diversity and prevalence of Atypical Porcine Pestivirus in the Midwest of US swine herds during 2016-2018. Transbound. Emerg. Dis..

[bib0061] Zhao D., Yang B., Yuan X., Shen C., Zhang D., Shi X., Zhang T., Cui H., Yang J., Chen X., Hao Y., Zheng H., Zhang K., Liu X. (2021). Advanced research in porcine reproductive and respiratory syndrome virus co-infection with other pathogens in swine. Front. Vet. Sci..

