# Supplementary material for: Enhancing the understanding of coinfection outcomes: Impact of natural atypical porcine pestivirus infection on porcine reproductive and respiratory syndrome in pigs
Source: Virus Res. 2024 Aug 1;348:199443. doi: 10.1016/j.virusres.2024.199443 (PMC11342287; doi:10.1016/j.virusres.2024.199443)
Supplement: Supplementary file 1 [file mmc1.docx]

**Supplementary Materials**

**Table S1.** Interstitial pneumonia and PRRSV Immunohistochemistry scoring criteria.

| Interstitial Pneumonia | | PRRSV Immunohistochemistry | |
| --- | --- | --- | --- |
| **Score** | **Interpretation** | **Score** | **Interpretation** |
| 0 | Negative | 0 | Negative |
| 1 | Mild focal | 1 | Positive- few positive cells |
| 2 | Mild multifocal | 2 | Positive- moderate positive cells |
| 3 | Moderate focal | 3 | Positive -large number of cells |
| 4 | Moderate multifocal |  |  |
| 5 | Severe focal |  |  |
| 6 | Severe multifocal |  |  |

**Table S2.** Statistical analysis of individual lung lobes by Kruskal Wallis and Dunn's test with multiple comparisons corrections.

| Lung Area | Chi-Squared | P-Value | Multiple Comparisons ID | Multiple Comparisons (z) | Adjusted P-Value |
| --- | --- | --- | --- | --- | --- |
| **Left Apical*** | **6.8940** | **0.0262** | Uninfected control vs APPV^-ve^/PRRSV^+ve^ | 0.000 | >0.9999 |
|  |  |  | Uninfected control vs APPV^+ve^/PRRSV^+ve^ | 1.750 | 0.2401 |
|  |  |  | **APPV^-ve^/PRRSV^+ve^ vs APPV^+ve^/PRRSV^+ve^** | **2.397** | **0.0496** |
| **Right Apical*** | **6.8940** | **0.0332** | Uninfected control vs APPV^-ve^/PRRSV^+ve^ | 0.000 | >0.9999 |
|  |  |  | Uninfected control vs APPV^+ve^/PRRSV^+ve^ | 1.750 | 0.2401 |
|  |  |  | **APPV^-ve^/PRRSV^+ve^ vs APPV^+ve^/PRRSV^+ve^** | **2.397** | **0.0496** |
| Left Cardiac | 3.5000 | 0.2115 | Uninfected control vs APPV^-ve^/PRRSV^+ve^ | 0.000 | >0.9999 |
|  |  |  | Uninfected control vs APPV^+ve^/PRRSV^+ve^ | 1.247 | 0.6370 |
|  |  |  | APPV^-ve^/PRRSV^+ve^ vs APPV^+ve^/PRRSV^+ve^ | 1.708 | 0.2630 |
| Right Cardiac | 3.7410 | 0.1756 | Uninfected control vs APPV^-ve^/PRRSV^+ve^ | 0.5132 | >0.9999 |
|  |  |  | Uninfected control vs APPV^+ve^/PRRSV^+ve^ | 1.626 | 0.3120 |
|  |  |  | APPV^-ve^/PRRSV^+ve^ vs APPV^+ve^/PRRSV^+ve^ | 1.493 | 0.4064 |
| **Intermediate*** | **6.8940** | **0.0332** | Uninfected control vs APPV^-ve^/PRRSV^+ve^ | 0.000 | >0.9999 |
|  |  |  | Uninfected control vs APPV^+ve^/PRRSV^+ve^ | 1.750 | 0.2401 |
|  |  |  | **APPV^-ve^/PRRSV^+ve^ vs APPV^+ve^/PRRSV^+ve^** | **2.397** | **0.0496** |
| Left  Diaphragmatic | 2.9460 | 0.2384 | Uninfected control vs APPV^-ve^/PRRSV^+ve^ | 0.8259 | >0.9999 |
|  |  |  | Uninfected control vs APPV^+ve^/PRRSV^+ve^ | 1.619 | 0.32 |
|  |  |  | APPV^-ve^/PRRSV^+ve^ vs APPV^+ve^/PRRSV^+ve^ | 1.036 | 0.90 |
| Right  Diaphragmatic | 4.8470 | 0.0668 | Uninfected control vs APPV^-ve^/PRRSV^+ve^ | 0.5534 | >0.9999 |
|  |  |  | Uninfected control vs APPV^+ve^/PRRSV^+ve^ | 1.833 | 0.2003 |
|  |  |  | APPV^-ve^/PRRSV^+ve^ vs APPV^+ve^/PRRSV^+ve^ | 1.719 | 0.2566 |
| Total Lung | 4.3630 | 0.0997 | Uninfected control vs APPV^-ve^/PRRSV^+ve^ | 0.8029 | >0.9999 |
|  |  |  | Uninfected control vs APPV^+ve^/PRRSV^+ve^ | 1.884 | 0.1787 |
|  |  |  | APPV^-ve^/PRRSV^+ve^ vs APPV^+ve^/PRRSV^+ve^ | 1.433 | 0.4559 |

*Indicates significance (P<0.05).

**Table S3.** Interstitial pneumonia and PRRSV immunohistochemistry analysis for the right lung lobe.

| Group | ID | Immunohistochemistry Score | Interstitial Pneumonia Score |
| --- | --- | --- | --- |
| APPV^-ve^/PRRSV^-ve^ | 545 | 0 | 0 |
| APPV^-ve^/PRRSV^-ve^ | 553 | 0 | 1 |
| APPV^-ve^/PRRSV^+ve^ | 510 | 0 | 3 |
| APPV^-ve^/PRRSV^+ve^ | 514 | 1 | 5 |
| APPV^-ve^/PRRSV^+ve^ | 503 | 0 | 4 |
| APPV^-ve^/PRRSV^+ve^ | 506 | 1 | 4 |
| APPV^-ve^/PRRSV^+ve^ | 542 | 0 | 2 |
| APPV^+ve^/PRRSV^+ve^ | 935 | 0 | 6 |
| APPV^+ve^/PRRSV^+ve^ | 939 | 0 | 3 |
| APPV^+ve^/PRRSV^+ve^ | 933 | 0 | 5 |
| APPV^+ve^/PRRSV^+ve^ | 938 | 3 | 5 |
| APPV^+ve^/PRRSV^+ve^ | 928 | 2 | 4 |
| APPV^+ve^/PRRSV^+ve^ | 932 | 1 | 4 |
| APPV^+ve^/PRRSV^+ve^ | 948 | 2 | 6 |
